# Identifying the Factors Related to Depressive Symptoms Amongst Community-Dwelling Older Adults with Mild Cognitive Impairment

**DOI:** 10.3390/ijerph16183449

**Published:** 2019-09-17

**Authors:** Dan Song, Doris S.F. Yu, Polly W.C. Li, Qiuhua Sun

**Affiliations:** 1 School of Nursing, Zhejiang Chinese Medical University, Hangzhou 310053, China; songdan@zcmu.edu.cn; 2 The Nethersole School of Nursing, The Chinese University of Hong Kong, Hong Kong, China

**Keywords:** mild cognitive impairment, depressive symptoms, correlates

## Abstract

High-level depressive symptoms have been reported in individuals with mild cognitive impairment (MCI), resulting in increased risk of progression to dementia. However, studies investigating the correlates of depressive symptoms among this population are scarce. This study aimed to investigate the significant socio-demographic, lifestyle-related and disease-related correlates of depressive symptoms among this cohort. Cross-sectional data were obtained from a sample of 154 Chinese community-dwelling older adults with MCI. MCI subjects were screened by the Montreal Cognitive Assessment. Depressive symptoms were measured by the Geriatric Depression Scale. Possible correlates of depressive symptoms in individuals with MCI were explored by multiple linear regressions. The prevalence of depressive symptoms among Chinese older adults with MCI was 31.8%. In multiple regression analysis, poor perceived positive social interaction, small social network, low level of physical activity, poor functional status, subjective memory complaint, and poor health perception were correlated with depressive symptoms. The findings highlight that depressive symptoms are sufficient to warrant evaluation and management in older adults with MCI. Addressing social isolation, assisting this vulnerable group in functional and physical activities, and cultivating a positive perception towards cognitive and physical health are highly prioritized treatment targets among individuals with MCI.

## 1. Introduction

Along with rapid global population ageing, the number of older adults afflicted with cognitive impairment is increasing dramatically [1]. Mild cognitive impairment (MCI) represents a precursor of dementia, wherein cognitive impairment is beyond that seen in normal age but does not reach dementia [2]. Community-based prevalence of MCI ranges from 16% to 22.2% worldwide [3,4,5]. Individuals with MCI represent a high-risk group for developing dementia [2]. Approximately 10% of individuals with MCI are estimated to convert to dementia annually, whereas the annual conversion rate to dementia is approximately 1–3% in overall older adults [6,7]. The high prevalence, progression rate to dementia, and considerable healthcare expenditure have rendered MCI as a major public health concern.

Neuropsychiatric symptoms are common amongst individuals with MCI [8]. Amongst them, depressive symptoms are predominant, with the reported prevalence ranging from 22.3% to 63.3% [9]. Though highly prevalent, depressive symptoms commonly go neglected in this population. This is mainly due to the fact that depressive symptoms are traditionally viewed as confounders in the detection of “true” cognitive impairment and, as such, are often treated as exclusion criteria in studying MCI. Recently, increasing evidence suggests that depressive symptoms, in fact, strongly and independently expedite the progression from MCI to dementia. Prospective studies indicate that depressive symptoms implicate a two- to four-fold increase in the risk of progression to dementia in individuals with MCI [10,11]. The consequences of depressive symptoms on the quality of life of individuals with MCI and their families are also substantial [12,13]. Therefore, depressive symptoms deserve more attention in the care of MCI.

In the current literature, no intervention studies targeting depressive symptoms for individuals with MCI exist. The majority of individuals with MCI live in the community [14]. Research is urgently needed to help guide community healthcare practice in reducing depressive symptoms amongst this clinical cohort. A key step in designing interventions for depressive symptoms in MCI is to identify the correlated factors. Literature from nursing, geriatric medicine, and psychology suggests that not only disease-related but also socio-demographic, lifestyle-related, and environment-related factors may affect the likelihood of psychopathology [15,16,17,18,19]. However, few studies have provided a comprehensive analysis on the factors associated with depressive symptoms in MCI. The focus has been placed on identifying the independent relationship of depressive symptoms with either demographic or disease-related variables.

Amongst all variables, social support and social network confer protection from depressive symptoms [20]. Various types of social support may exert different effects under different situations. Emotional support reduces depressive symptoms amongst healthy older adults [21], and tangible support reduces depressive symptoms amongst older adults with clinical diseases [22]. A precise understanding of the role of social support in affecting depressive symptoms amongst individuals with MCI is needed to assist those patients to cope with stress associated with the disease.

The relationship between disease-related factors and depressive symptoms is unclear. The study by Chan et al. [19] failed to identify the association between objective cognitive function and depressive symptoms in MCI. By contrast, subjective cognitive complaint exhibits an evident relationship with depressive symptoms amongst older adults [23]. Functional ability and health status appeared to have a more evident relationship with depressive symptoms when they were measured using subjective rather than objective methods [24,25].

Amongst the lifestyle-related factors, physical activity relieves stress, boosts self-efficacy, and is consistently associated with good mood status in general older population [26,27]. However, the role of physical activity in influencing depressive symptoms amongst individuals with MCI has seldom been studied. Amongst the demographic variables, old age, being female, low education level, low income, and being single expose general older adults to high emotional disturbance [28,29], and their effects are less conclusive for older adults with MCI [18,19].

A thorough understanding of the correlates of depressive symptoms amongst community-dwelling older adults with MCI is needed to assist community healthcare providers to identify individuals with MCI who are at risk of developing depressive symptoms and provide guidance for interventions to ameliorate depressive symptoms in MCI. Hence, the objective of the present study is to provide a comprehensive analysis on the socio-demographic, disease-related, and lifestyle-related factors associated with depressive symptoms in community-dwelling older adults with MCI.

## 2. Materials and Methods

### 2.1. Ethics Statement

This study was approved by the Survey and Behavioral Research Ethics Committee of the Chinese University of Hong Kong (No. SBREC-20160602). Prior to the data collection, each participant signed a consent form that detailed the nature of the study. The participants were also assured of the confidentiality of their responses.

### 2.2. Study Design and Sample

A cross-sectional study design was used. Eligible participants were community-dwelling older adults aged over 60 who were screened with MCI. MCI was defined by scoring 19–26 on the Montreal Cognitive Assessment (Chinese version, MoCA-C) [30]. MoCA is a tool specifically developed for screening MCI [31]. By using a cut-off score of 19 and 26, the MoCA-C gives the optimal sensitivity and specificity in differentiating individuals with MCI from those with dementia (sensitivity: 93.2% and specificity: 71.7%) and intact cognitive function (sensitivity: 92.4% and specificity: 88.4%), respectively [30]. The influence of education on cognitive function was adjusted by adding one point to those with less than 6 years of education [32]. Participants were excluded if they were taking anti-depressant agents, had any neurological disorders that influenced cognition (e.g., stroke, Parkinson’s disease, or head damage), or had impaired hearing or vision that may have inhibited them from giving consent and answering the questionnaires. Finally, a convenient sample of 154 community-dwelling older adults with MCI was recruited.

Regarding sample size estimation, researchers conservatively estimated a medium effect size (R^2^ = 0.13) of the relationship between the sleep quality and the group of candidate correlates [33]. A total of 14 independent variables were considered to be included into the multivariate analysis; thus, a minimum of 135 participants were required in this study.

### 2.3. Data Collection and Measurements

Data collection took place in a public community healthcare center in the city of Hangzhou, Southeast China. Participants were consecutively recruited when they were visiting the healthcare center for their annual health check-ups between June 2016 and July 2017.

Three registered nurses collected the data using paper and pencil method. A number of validated tools were used to measure the criterion variable of depressive symptoms and socio-demographic, disease-related, and lifestyle-related correlates of depressive symptoms. 

Depressive symptoms were assessed by the 30-item Geriatric Depression Scale (Chinese version, GDS-C) [34]. The scale results of using dichotomous questions presented a total score ranging from 0 to 30, with high scores representing highly depressive symptoms. Good internal consistency (Cronbach’s α = 0.846) and construct validity of GDS-C were confirmed [34]. A score ≥ 10 indicates presence of suggested clinical depression [35].

Socio-demographic data included age, gender, education, marital status, income, living conditions, social network size, and social support. Social support was assessed by the 20-item Medical Outcomes Study Social Support Survey (Chinese version; MOS-SSS-C) [36]. The first item in the MOS-SSS-C measures the size of social network by asking the respondents to indicate the number of their close friends and relatives. The 19 other items are divided into four subscales to measure the four dimensions of social support: (1) emotional and informational support, (2) tangible support, (3) positive social interaction, and (4) affectionate support. The subscale score is the sum of responses checked for the relevant items and rescaled to a 0–100 range, with a high score representing high perceived social support. Satisfactory internal consistency (Cronbach’s *α* = 0.98), test-retest reliability (*r* = 0.84), and construct validity were reported [36].

Physical activity level was measured by the International Physical Activity Questionnaires short version (Chinese version, IPAQ-SF-C). The IPAQ-SF classifies the level of physical activity into three groups: Low, moderate and high. Criterion validity was established by the significant correlation with the pedometer-measured steps [37].

Disease-related factors included objective cognitive function, subjective memory compliant, chronic disease condition, perceived health status, and functional status. Objective cognitive function was reflected by MoCA score. Subjective memory complaint was measured by the Memory Inventory for Chinese (MIC) [38]. The questionnaire consists of 27 questions that explore perceived memory problems in daily life. The total score ranges from 0 to 108, with a high score representing considerable awareness of memory limitations. The MIC shows good internal consistency (Cronbach’s α = 0.89) and construct validity [38]. Burden of comorbidities were determined from the medical records of patients and quantified by the Charlson Comorbidity Index [39] to indicate the weight of overall chronic diseases. Perceived health status was indicated by EuroQoL visual analogue scale (0–100), with high scores indicating high self-rated health [40]. Functional status was assessed by Functional Activities Questionnaire (Chinese version, FAQ-C). FAQ evaluates complex functional and social behaviors of older adults that are probably impaired during early cognitive decline stage, and is considered as more sensitive than the Lawton and Brody’s IADL scale (0.85 vs. 0.57) in detecting functional impairment in those with early cognitive impairment [41].

### 2.4. Statistical Analyses

Statistical analysis was performed with SPSS version 22 (SPSS Inc., Chicago, IL, USA). Correlation analysis was conducted to examine significant bivariate associations between all the dependent and independent variables, using the Pearson’s correlation and Spearman’s Rho for the continuous and ordinal variables, respectively. As for the nominal potential correlates of gender, marital status, and living condition, independent *t*-tests were used to examine for any significant difference in the Geriatric Depression Scale (GDS) score between their respective dichotomous groups. Variables that were significantly associated with depressive symptoms in the bivariate analyses were then entered into the multiple regression models with a backward selection mode to retain the significant ones in the final regression model obtained. Regression diagnostics were performed to determine whether relevant statistical assumptions were met. We also performed the following sensitivity analysis to determine the stability of the final regression model: (1) participants aged above 85 were removed from the analysis; (2) logistic regression analysis was conducted with binary dependent variable (clinical level of depressive symptoms vs. no clinical level of depressive symptoms). The significance level α was set at 0.05, and all comparisons were two tailed.

## 3. Results

### 3.1. Characteristics of the Participants

A total of 527 older adults were approached for investigation. Amongst them, 121 declined to participate. The response rate was 77%. A total of 406 adults were screened for eligibility, with 51 excluded for not satisfying the inclusion criteria. Amongst the 355 participants who completed the survey, 154 satisfied the criteria for MCI. No missing data existed. The descriptive characteristics of the participants are summarized in Table 1.

The sample was mostly composed of women (74.7%) and well advanced in age, with a mean of 75.6 years. Fewer than 30% of participants were single, and 16.2% of participants lived alone. Fewer than 20% of participants indicated an education level above middle school, and more than 70% of participants received a monthly income that was under the average level in a local city (4000 CNY). Nearly a quarter of the participants presented a low level of physical activity. The average social network size was six. Amongst the different types of social support, the participants perceived tangible and affectionate support as adequate. Nearly half of the participants indicated the presence of functional impairment. Comorbidities were common. The mean Montreal Cognitive Assessment score was 23.08, and the mean MIC score was 7.25. The participants reported a rather high level of depressive symptoms, and the mean Geriatric Depression Scale (GDS) score was 6.79. Using a cut-off score of 10, nearly one third of the participants (31.8%) were suspected with suggested clinical depression.

### 3.2. Bivariate Correlations between Depressive Symptoms and Candidate Correlates

Amongst the 15 potential continuous or ordinal correlates, seven of them showed significant correlation with the GDS score. Table 2 presents the correlation matrix. A higher level of depressive symptoms was correlated significantly with lower income, smaller social network, lesser tangible social support, lesser positive social interaction, more functional difficulties, lower level of physical activity, and more subjective memory complaints. As for the three nominal independent variables, namely, gender, marital status, and living arrangement, GDS score did not differ between their respective dichotomous groups. The seven variables that were found to be significantly related to the GDS score were then entered to the multiple regression model.

### 3.3. Multivariate Correlates of Depressive Symptoms amongst Individuals with MCI

The variance inflation factor of the model ranges from 1.024–1.638, indicating no presence of multicollinearity. Table 3 presents the results of the final multiple regression model. In the final model, six variables showed significant contribution to the variance of the GDS score. These variables were social network (standardized *β* = −0.138, *p* = 0.032), positive social interaction (standardized *β* = −0.278, *p* < 0.001), subjective memory complaint (standardized *β* = 0.202, *p* = 0.008), functional impairment (standardized *β* = 0.225, *p* = 0.004), perceived health status (standardized *β* = −0.264, *p* < 0.001), and level of physical activity (standardized *β* = −0.154, *p* = 0.014). The final model significantly accounted for 46.5% of the variance of GDS score [with *F* (1146) = 21.35, *p* < 0.001].

### 3.4. Sensitivity Analysis of the Multiple Regression Model

A sensitivity analysis was performed, excluding participants aged above 85, with the regression results remaining unchanged. The other sensitivity analysis was conducted by turning the linear regression model into logistic regression model, the results of the logistic regression model showed that all the significant correlates of depressive symptoms in the linear regression model remained significant in the logistic regression model, except for the level of physical activity (Supplementary Table S1). The results of the sensitivity analysis indicated the stability of the results of the regression model.

## 4. Discussion

### 4.1. Interpretation of the Study Findings

This is the first study in Mainland China to examine the manifestation and predictors of depressive symptoms in older adults with MCI. The result showed that a large proportion of Chinese community-dwelling older adults with MCI (31.8%) experienced suggested clinical level of depressive symptoms; this finding concurred with a systematic review on the worldwide prevalence of depressive symptoms in MCI, which reported a pooled prevalence of 32% [9]. This study is different from previous work in that the influence of socio-demographic, disease-related, and lifestyle-related factors on depressive symptoms in MCI was examined simultaneously. The findings of this study suggest that the level of depressive symptoms in MCI is not only associated with disease-related factors, but social and lifestyle-related factors also play an important role in depression severity in MCI.

We found that less social interaction and smaller social network in individuals with MCI are significantly correlated with higher level of depressive symptoms, which is concurred with the well-hypothesized buffering role of social support in coping with stress [42]. Amongst the various functional types of social support, positive social interaction demonstrated the strongest correlation with depressive symptoms amongst individuals with MCI. Given that this dimension is defined by the availability of other persons to share pleasurable and relaxed activities and make one’s mind relaxed for a while [43], positive social interaction may offer a sense of social belonging and integration and enable individuals with MCI to relieve from the distress of functional and cognitive decline, thus reducing depressive symptoms [44]; however, other types of social support demonstrated no association with depressive symptoms. This finding may be partly due to the sample demographic characteristics. The majority of the participants lived with their family. In Chinese culture, family members play a central role in providing tangible, emotional, and affectionate support [45]. Nevertheless, the amount of positive social interaction relies largely on one’s social network beyond family members. This finding further highlighted the importance of social network and face-to-face social interaction in determining the psychological well-being of older adults with MCI.

Amongst the disease-related factors, a significant association was found between depressive symptoms and subjective measure of cognitive, physical, and functional fitness. However, no association was found between depressive symptoms and objective measure of cognitive function or comorbidity illness. This finding is in line with previous studies [23,25], suggesting that the subjective measure of the disease impact is more relevant to psychological health than the objective measure. This situation echoes with the definition of stress, which is maintained as a relationship between the environment that is appraised by the person as taxing or exceeding his or her resources [46]. Thus, objective cognitive and physical difficulties experienced by the individuals with MCI might not be appraised as stressful per se, if persons have sufficient recourse (e.g., social support) for coping with these difficulties. This is supported by the results of the bivariate correlation of this study, which indicated that the subjective measure of cognitive and health status was not only moderately associated with objective cognitive function and physical status but also associated with social support. However, objective cognitive function and physical status were not significantly related to social support.

The significant role of low-level physical activity in accounting for the depressive symptoms of individuals with MCI concurred with numerous studies that reported the positive effects of physical activity on mood [26,27]. Physical activity stimulates brain chemicals, such as serotonin and dopamine, which play an important role in improving the mood and overall sense of well-being [47]. In addition, the social withdrawal hypothesis suggested that increased sedentary time may remove individuals from social interactions and thereby increase their risk for depression.

The other socio-demographic factors (age, gender, income, education, residence, marriage, and stressful life event) demonstrated no association with depressive symptoms in MCI. These variables were correlates in some studies [28,48] but not in others [49,50]. The difference was probably due to the variation in controlling confounding factors, inconsistency of the evaluation methods, sample size, and characteristics.

### 4.2. Implications

Depressive symptoms are prominent enough to warrant evaluation and treatment for community-dwelling older adults with MCI. With regard to the detection of depressive symptoms, the evident relationship between depressive symptoms and subjective measure of disease-related factors (cognitive function, health status, and functional impairment) underscores the importance of incorporating subjective assessment of disease-related factors into the identification of individuals with MCI who are at risk of developing depressive symptoms. Moreover, considerable attention should be paid to individuals with MCI who are socially isolated or physically inactive as they are at a higher risk of developing depressive symptoms.

With regard to the management of depressive symptoms in MCI, in light of the strong correlation between social network, social support and depressive symptoms, expanding one’s social network and continuing social engagement are crucial. To facilitate social engagement amongst individuals with MCI, social activities should be designed as meaningful and enjoyable to motivate older adults with MCI to participate. Low-level physical activity was also identified to be associated with a high level of depressive symptoms. Thus, encouraging individuals with MCI to engage actively in physical activities may be effective to reduce depressive symptoms in MCI. Group-based exercise interventions provide an excellent platform for promoting physical activity and social interaction [51] and can be considered to be applied to community-dwelling older adults with MCI.

### 4.3. Limitations

This study displays several methodological limitations. Firstly, the cross-sectional study design limits the strength of the study in making casual inference on the effect of the identified correlates on depressive symptoms. Secondly, this study mainly focused on individual-level factors and ignored environmental-level variables, such as neighborhood social capital and neighborhood greenness, which may be associated with depressive symptoms. The incorporation of such variables into this study will allow a thorough examination of the correlates of depressive symptoms in individuals with MCI. Thirdly, the MCI classification was based on a screening tool and not on a clinical diagnosis, which should be noted when comparing our results with those of studies based on clinically confirmed samples.

## 5. Conclusions

This community-based study found that the prevalence of depressive symptoms is as high as 31.8% in Chinese older adults with MCI. The correlates of depressive symptoms identified in this study highlighted the importance of addressing social support and encouraging physical activity for the psychological well-being of community-dwelling older adults with MCI.

## Figures and Tables

**Table 1:** Characteristics of the participants (*N* = 154).

Characteristics	Values
**Socio-demographic factors**	
Age ^a^	75.6 ± 7.40
Gender ^b^	
Male	39 (25.3%)
Female	115 (74.7%)
Marital status ^b^	
Married	109 (70.8%)
Single	45 (29.2%)
Education level ^b^	
Below middle school	69 (44.8%)
Middle school and above	85 (55.2%)
Residence ^b^	
Living alone	25 (16.2%)
Living with others	129 (83.8%)
Monthly income ^c^	
Less than 4000 CNY	110 (71.4%)
Above 4000 CNY	44 (28.6%)
Size of social network	6.63 ± 2.88
Perceived social support (MOS-SSS-C score) ^a^	
Overall	75.61 ± 8.51
Tangible subscale	94.79 ± 5.66
Affectionate subscale	76.41 ± 8.97
Emotional and information subscale	66.18 ± 12.52
Positive social interaction subscale	65.07 ± 10.37
**Lifestyle-related factors**	
Physical activity level (IPAQ grading)	
Low	33 (21.4%)
Moderate/high	121 (78.6%)
**Disease-related factors**	
Burden of comorbidities (CCI score) Number of comorbidity ^a^	1.86 ± 1.09
0	9 (5.8%)
1	56 (36.4%)
2	51 (33.1%)
3	28 (18.2%)
4	8 (5.2%)
5	1 (0.6%)
6	1 (0.6%)
Perceived health status (EQ-VAS) ^a^	75.62 ± 10.04
Objective cognition (MoCA score) ^a^	23.08 ± 1.86
Subjective memory complaint (MIC score) ^a^	7.25 ± 4.21
Functional status (FAQ score) ^a^	0.83 ± 1.01
0	78 (50.6%)
1	44 (28.6%)
2	19 (12.3%)
3	9 (5.8%)
4	1 (0.6%)
5	3 (1.9%)
**Depressive symptoms**	
GDS score	6.79 ± 4.01
Presence of suggested clinical depression ^b^	49 (31.8%)

Note: CNY = Chinese yuan, CCI = Charlson comorbidity index, EQ-VAS = EuroQol-Visual Analogue Scale, MOS-SSS-C, Chinese version of Medical Outcomes Study Social Support Survey, MoCA = Montreal Cognitive Assessment, MIC = Memory inventory for the Chinese, FAQ = Functional Activities Questionnaire, IPAQ = International Physical Activity Questionnaire. ^a^ Mean ± S.D. ^b^ *N* (%). ^c^ 1 U.S. Dollar = 6.9 CNY.

**Table 2:** Correlations of socio-demographic features, lifestyle factors, disease-related factors, and depressive symptoms in MCI (*N* = 154).

No.	Variable	1	2	3	4	5	6	7	8	9	10	11	12	13	14	15
1	GDS	1.000														
2	Age	0.0404	1.000													
3	Income	−0.159 *	0.336 **	1.000												
4	Education	−0.154	−0.187 *	−0.418 **	1.000											
5	Size of social network	−0.257 **	0.097	−0.086 *	−0.045	1.000		.								
6	MOS-SSS-C tangible	−0.422 **	−0.041	0.142	0.113	0.225 **	1.000									
7	MOS-SSS-C affectionate	−0.073	−0.012	−0.001	0.013	0.228 **	0.460 **	1.000								
8	MOS-SSS-C emotional-information	0.113	0.061	−0.159 *	−0.190 *	0.007	0.167 *	0.286 **	1.000							
9	MOS-SSS-C positive social interaction	−0.293 **	−0.111	0.115	0.131	0.182 *	0.474 **	0.486 **	0.051	1.000						
10	FAQ	0.500 **	0.168 *	−0.113	−0.192 *	−0.074	−0.248 **	0.155	0.136	−0.016	1.000					
11	EQ-VAS	−0.447 **	−0.046	0.019	−0.022	0.255 **	0.058	−0.152	−0.101	0.035	−0.365 **	1.000				
12	CCI	0.031	0.020	0.059	−0.073	0.045	−0.089	0.030	0.029	0.018	−0.025	−0.195 *	1.000			
13	IPAQ	−0.262 **	−0.039	−0.066	0.020	0.016	0.224 **	0.032	0.099	0.063	−0.197 *	0.087	−0.009	1.000		
14	MIC	0.406 **	0.071	−0.120	0.235 **	0.042	−0.042	0.172 *	0.170 *	0.058	0.482 **	−0.266 **	−0.068	−0.117	1.000	
15	MoCA	−0.113	−0.369 **	0.078	−0.090 **	−0.024	0.045	−0.045	−0.148	−0.003	−0.215 **	0.052	0.062	0.170 *	−0.217 **	1.000

Note: MIC = Memory Inventory for Chinese, CCI = Charlson comorbidity index, GDS-C = Chinese version of Geriatric Depression Scale, MoCA-C = Chinese version of Montreal Cognitive Assessment, CCI = Charlson Comorbidity Index, MOS-SSS-C, Chinese version of Medical Outcomes Study Social Support Survey, EQ-VAS = EuroQol-Visual Analogue Scale, IPAQ = International Physical Activity Questionnaire. * *p* < 0.05, ** *p* < 0.01.

**Table 3:** Multiple regression analysis for the correlates of the GDS-C score in patients with MCI (*N* = 154).

Variable	*β* (95% CI)	SE	Standardized *β*	*p* Value
Social network	−0.192 (−0.367, −0.016)	0.089	−0.138	0.032
MOS-SSS-C (positive social interaction)	−0.103 (−0.148, −0.057)	0.023	−0.278	<0.001
MIC	0.192 (0.052, 0.333)	0.071	0.202	0.008
FAQ	0.818 (0.267, 1.370)	0.279	0.225	0.004
EQ-VAS	−0.105 (−0.151, −0.047)	0.027	−0.264	<0.001
IPAQ	−1.502 (−2.701, 0.303)	0.607	−0.154	0.014

Note: *β* = regression coefficient, 95% CI = 95% Confidential Interval, MOS-SSS-C, Chinese version of Medical Outcomes Study Social Support Survey, EQ-VAS = EuroQol-visual Analogue Scale, FAQ = Functional Activities Questionnaire, MIC = Memory Inventory for Chinese, IPAQ = International Physical Activity Questionnaire.

## Supplementary Materials

The following are available online at https://www.mdpi.com/1660-4601/16/18/3449/s1, Table S1: Logistic regression analysis for the correlates of the depressive symptoms in patients with MCI.
