# Supplementary material for: Identifying the Factors Related to Depressive Symptoms Amongst Community-Dwelling Older Adults with Mild Cognitive Impairment
Source: Int J Environ Res Public Health. 2019 Sep 17;16(18):3449. doi: 10.3390/ijerph16183449 (PMC6765998; doi:10.3390/ijerph16183449)
Supplement: Supplementary file 1 [file ijerph-16-03449-s001.pdf]

Supplementary Table 1. Logistic regression analysis for the correlates of the depressive symptoms in patients with MCI (*N* = 154).

Variable	OR	95% CI	P value
Social network	0.804	0.667–0.970	0.022
MOS-SSS-C (positive social interaction)	0.956	0.919–0.994	0.024
MIC	1.134	1.006–1.278	0.40
FAQ	1.721	1.116–2.654	0.014
EQ-VAS	0.946	0.904–0.991	0.018

Note: OR = odds ratio, 95% CI = 95% Confidential Interval, MOS-SSS-C, Chinese version of Medical Outcomes Study Social Support Survey, MIC = Memory Inventory for Chinese, EQ-VAS = EuroQol-visual Analogue Scale, FAQ = Functional Activities Questionnaire.
